# Efficacy of a Chinese Herbal Medicine Compound Zhangpi Ointment against Hydroxyurea-Induced Leg Ulcers: A Prospective, Randomized, Open-Label, Controlled Clinical Trial

**DOI:** 10.1155/2018/9329465

**Published:** 2018-12-12

**Authors:** Yu-yang Pang, Yan Li, Gang Kui, Yong Tang, Ming-juan Liao, Yong-ling Wang, Zhen-dong Cao, Qi Zhu

**Affiliations:** ^1^Department of Hematology, Shanghai Ninth People's Hospital, Shanghai Jiao Tong University School of Medicine, Shanghai 200011, China; ^2^Department of Traditional Chinese Medicine, Shanghai Ninth People's Hospital, Shanghai Jiao Tong University School of Medicine, Shanghai 200011, China

## Abstract

*Objective. *The randomized controlled trial was to evaluate the efficacy of topical Chinese herbal Zhangpi Ointment for hydroxyurea-induced leg ulcers in patients with myeloproliferative neoplasms.* Patients and Methods. *This single-center, prospective, randomized, open-label, controlled clinical trial conducted at Shanghai Ninth People's Hospital enrolled 54 patients with hydroxyurea-induced leg ulcers. Patients were randomly assigned to the control group (n = 27) treated with chlorhexidine dressing or the intervention group (n = 27) treated with the Zhangpi Ointment. Finally, 26 patients in the control group and 23 patients in the intervention group completed 8 weeks of observation.* Results. *The rate of complete healing was 100% for the intervention group, which was significantly higher than that of the control group (96.15%) (*P*<0.05). Furthermore, the intervention group achieved a significantly higher rate of wound healing (95.56%) than the control group (69.02%) at week 4 (*P*<0.01). The intervention group took 34 ± 5 days to achieve complete healing while the control group took 41 ± 7 days (*P *< 0.01). Moreover, grade 3/4 side effects were observed in neither group.* Conclusion. *The Zhangpi Ointment is effective in promoting the healing of hydroxyurea-induced leg ulcers in patients with myeloproliferative neoplasms, providing a therapeutic option for a condition that is recalcitrant to conventional therapy.

## 1. Introduction

Myeloproliferative neoplasms (MPNs) are a group of stem cell disorders characterized by clonal myeloproliferation that is devoid of dyserythropoiesis, granulocytic dysplasia, or monocytosis. Hydroxyurea (HU) is an S-phase specific chemotherapeutic agent which inhibits DNA synthesis through its action on ribonucleotide diphosphate reductase [1] and is widely used in the treatment of MPNs. Although HU is relatively well tolerated, adverse effects often occur in 9% of treated patients [2, 3]. Rare and severe adverse effects appear to be associated with long-term administration and may be systemic or restricted to skin and mucous membranes [4]. The occurrence of painful leg ulcers represents another rare and incompletely characterized complication that has been described in patients with myeloproliferative diseases receiving high-dose (mean dose 28.2 mg/kg) and long-term (mean treatment duration 39 months) HU treatment [5, 6]. This cutaneous adverse effect is often treatment-resistant, causing delay in healing of the lesion (mean healing duration 4.3 months) [7]. Neovascularization and circulation deficiency and macroerythrocytosis during hydroxyurea treatment may be involved in the development of these rare ulcers, via impairment of the microcirculatory rheology [8]. Poor response to traditional local and systemic therapy is a typical feature of HU-induced leg ulcers, and discontinuation of the drug is often required to achieve complete wound healing [9, 10]. Although discontinuation of treatment is still the option of choice for complete recovery [11], leg ulcer can recur even after discontinuation of HU treatment [12]. Chlorhexidine diacetate released from wound dressing was used as an antimicrobic drug for the treatment of wound including skin ulcers [13]. It has been reported that two patients were successfully treated with a kind of matrix metalloproteinase modulator which is able to modulate the activity of proteases [14] and one patient was successfully treated with a topical basic fibroblast growth factor product, leading to rapid improvement within 2 weeks, without adverse effects [15]. However, the sample size was too small.

Zhangpi Ointment (ZPO) is a mixture of six topical Chinese herbs and two natural minerals, which include* Glycyrrhiza uralensis Fisch*,* Angelica sinensis (Oliv.) Diels, Arnebia euchroma (Royle) Johnst., Rheum officinale Baill, Rehmannia glutinosa Libosch, Cortex Lycii*, rubber powder, and calomel. Licoflavone, a component of* Glycyrrhiza uralensis Fisch*, was reported to promote gastric mucosal ulcer healing in mice by regulating inflammatory mediators [16]. Gastric ulcer healing effect has also been described for* Angelica sinensis (Oliv.) Diels* and other herbs [17, 18]. These findings, however, were mostly based on the effects of the medicinal herbs on gastrointestinal mucosal ulcers and in rodent models. No studies have been carried out on the effects of these medicinal herbs on leg ulcers and in humans.

Our anecdotal clinical experience suggests that the ZPO may be effective in promoting healing of HU-induced leg ulcers. In the current prospective randomized controlled trial, we sought to investigate the efficacy of the ZPO for HU-induced leg ulcers in 54 patients with MPNs.

## 2. Materials and Methods

### 2.1. Trial Design and Participants

The study protocol was designed by experts from Department of Hematology and Traditional Chinese Medicine, Shanghai Ninth People's Hospital. Study approval was obtained from ethics committees at our hospital. They were responsible for the training and guidance of investigators and oversaw all aspects of coordination and data collection. Records of the allocation details were kept at our hospital for data verification and checking at monitoring visits. The study was undertaken in accordance with the Declaration of Helsinki and registered at http://www.chictr.org.cn (Clinical Trials. Gov ID: ChiCTR-INR-16009001).

This was a single-center, prospective, randomized, open-label, controlled clinical trial of ZPO versus chlorhexidine dressing for HU-induced leg ulcers in patients with MPNs. We recruited 71 patients from Shanghai Ninth People's Hospital between July 2016 and April 2018. Eligible patients were aged at least 18 years and no older than 70 years, had histologically or cytologically confirmed MPNs which were treated with HU, and had grade 1 to 3 leg ulcer (Wagner's classification) that had persisted for at least 2 months. Patients who had severe infection or autoimmune disease, severe liver, kidney, cardiovascular and mental diseases, diabetes, severe malnutrition, or history of bleeding disorder were excluded. Enrolled patients who had severe adverse events, who had poor compliance, and who asked for withdrawal were eliminated. All patients provided written informed consent.

The primary objective was to compare rate of complete healing with co-primary analyses of ulcer improvement (changes in the area of the ulcer) and healing time (number of days taken to achieve complete wound healing). Secondary endpoint was adverse events which were evaluated and graded according to Common Terminology Criteria for Adverse Events (NCICTCAE 4.0).

### 2.2. Procedures

Based on our preliminary experimental results, the rate of complete healing of the intervention group and control group at the fourth week was 95% and 64%, respectively. Accordingly, in this superiority trial, the sample size was 54 (with *α*= 0.05, two-tailed and *β*= 0.2). All eligible patients were randomly assigned on a 1:1 basis to receive either ZPO or chlorhexidine dressing with standard wound care. Randomization lists were generated through http://www.randomizer.org and held at our hospital.

The pharmacological properties of ZPO are shown in Table 1. ZPO was manufactured by the pharmacy of our hospital by the standard preparation method of traditional Chinese ointment medicine. The entire process was carried out in a bacteria-free environment and with strict quality control.

Standard wound care included local debridement of necrotic tissue or callus and dressing changes once daily until the wounds were completely healed [19]. All wounds were cleansed with sterile saline prior to assessment and dressing application. ZPO (about 1 mm thick) was applied to the surface of the ulcer which was then covered by a sterile dressing. For the control group, chlorhexidine dressing was applied to the surface of the ulcer directly. The duration of follow-up was 8 weeks and every patient was followed up once a week. Changes in the area of the ulcer (cm^2^) were assessed at each follow-up visit.

### 2.3. Statistical Analysis

Changes in the area of the ulcer (cm^2^) were measured by digital photography on day 0 and at each follow-up visit and calculated using the Image-Pro ® Plus 6.0 (Media Cybernetics, Inc., Bethesda, MD, USA ) analysis software. The rate of wound healing was calculated according to the following formula: area healed (%) = (initial area - the residual wound area) / initial area × 100%. The rate of complete healing was defined as the percentage of patients who had complete (100%) healing: complete healing (%) = number of patients who had complete healing / total number of patients × 100%. Pathological scar formation was assessed according to the Vancouver Scar Scale [20].

The efficacy statistical analyses were prespecified and followed the intention-to-treat principle. All patients with a baseline assessment and at least one post-baseline assessment were included in analyses. Furthermore, patients who were randomized, received at least one treatment, and were assessed for adverse events were included in adverse events analysis. Continuous variables were described in mean ± standard deviation and were compared between groups by using the Wilcoxon rank sum test or rank ANCOVA for baseline adjustments. Student's* t* test was used to compare proportions and also to test for heterogeneity within subgroups defined by baseline characteristics. A* P* value < 0.05 was considered statistically significant. All calculations were performed using SPSS 18.0 (SPSS Inc., Chicago, IL, USA).

## 3. Results

### 3.1. Participant Flow

**Figure 1:**
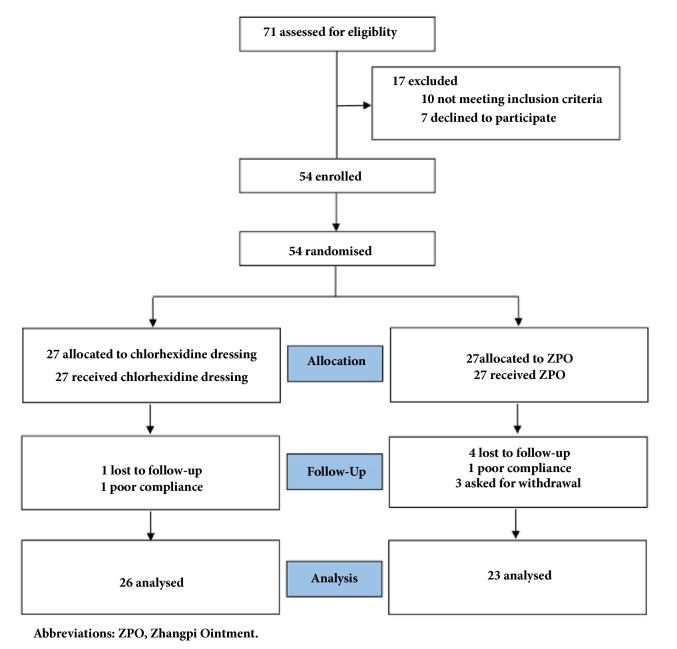
Flow of participants through the trial.

The study flowchart is shown in Figure 1. Seventy-one patients who developed leg ulcers after undergoing HU therapy were recruited. Ten patients who did not meet the eligibility criteria and 7 patients who declined participation were excluded. Finally, 54 patients were enrolled in this clinical trial and were randomly assigned on a 1:1 basis to receive either ZPO or chlorhexidine dressing. Two patients lost to follow-up due to poor compliance and three patients asked for withdrawal. Consequently, 49 patients were analysed.

### 3.2. Patient Demographic and Baseline Characteristics

The demographic and baseline characteristics of the study population are shown in Table 2. The median age of the study population was 44 (range, 24 to 60) years and 61.2% of the patients were male. Thirty-five (71.4%) patients were diagnosed with essential thrombocythemia, 9 (18.4%) with polycythemia vera, and 5 (10.2%) with myelofibrosis. Moreover, 59.2% of the patients had normal Body Mass Index (BMI). Each patient had one ulcer. The ulcers were evaluated according to the Wagner's classification and were classified to grade 1 (26.5%), grade 2 (42.9%), and grade 3 (30.6%). Mean ulcer area was 3.5±1.0 (cm^2^). We conducted germiculture of the ulcers for all patients and 18.4% of the patients received systemic antibiotic therapy due to staphylococcus aureus infection. Meanwhile, most of the patients suffered moderate (57.1%) and severe pain (28.6%). All patients received standard doses of HU (15-20 mg/kg, once daily), which was terminated and replaced with recombined human interferon *α* (3,000,000 units, three times a week) in 37 patients or watchful waiting in 12 patients when painful leg ulcers appeared. The median duration of ulcer prior to enrollment was 10 months. The two groups were comparable in demographic and baseline characteristics.

### 3.3. Outcomes and Estimation

**Figure 2:**
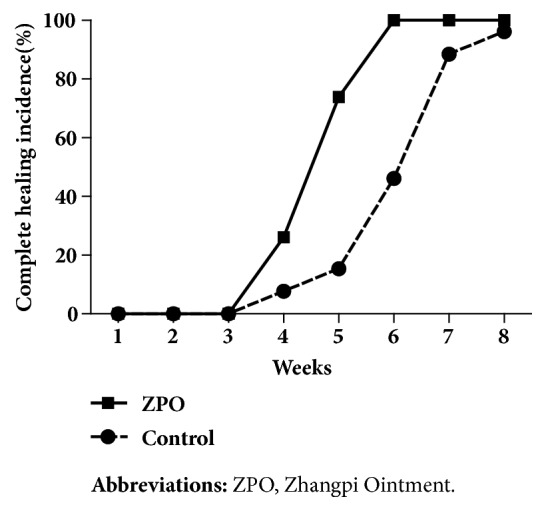
Complete healing incidence during the overall observation period of eight weeks.

**Figure 3:**
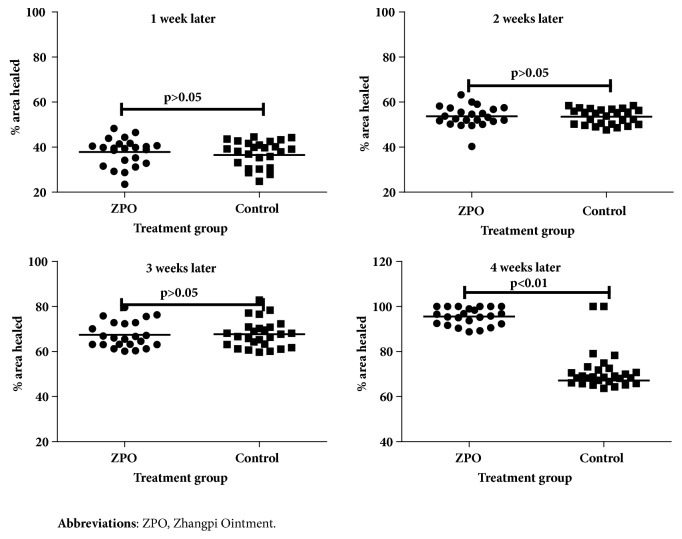
Mean % of ulcer area healed at each time point of assessment.

**Figure 4:**
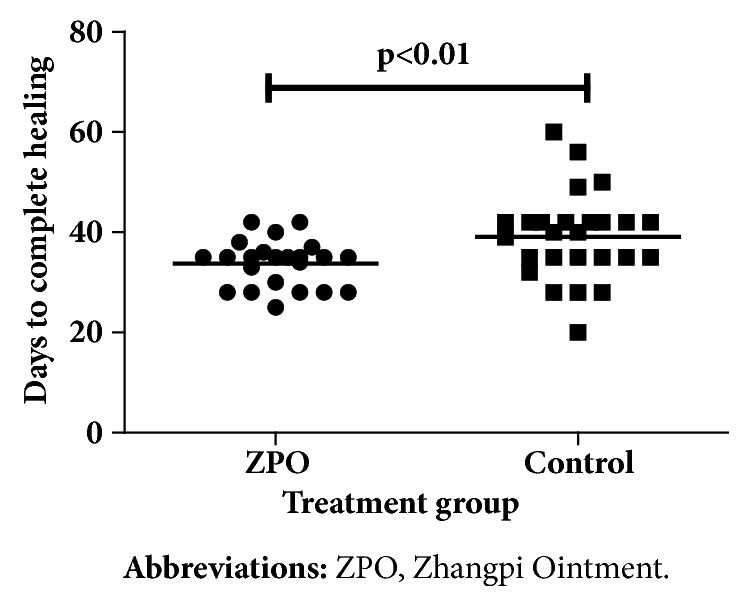
Days to complete ulcer healing of the two groups.

**Figure 5:**
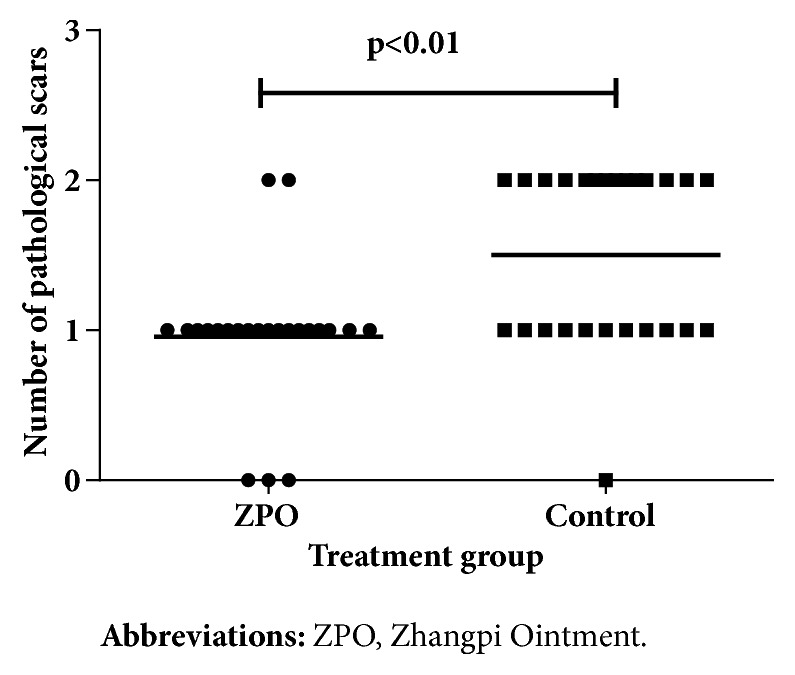
Pathological scar formation after complete healing for the two groups.

Forty-nine patients completed 8 weeks of treatment. The rate of complete healing was 100% for the intervention group, which was significantly higher than that of the control group (96.15%) (*P* < 0.05). Statistically significant difference in the rate of complete healing between the intervention group and the control group started to emerge at week 4 of treatment (intervention: 26.09% versus control: 6.90%;* P* < 0.05) (Figure 2). Furthermore, the intervention group achieved a significantly higher rate of wound healing (95.56%) than the control group (69.02%) at week 4 (*P* < 0.01) (Figure 3). The intervention group took 34 ± 5 days to achieve complete healing while the control group took 41 ±7 days (*P *< 0.01) (Figure 4). Moreover, the number of pathological scars was significantly smaller in the intervention group than the control group (*P* < 0.01) (Figure 5).

### 3.4. Adverse Events

Adverse events were evaluated and graded according to Common Terminology Criteria for Adverse Events (NCICTCAE 4.0).  Table 3 shows the frequency of common side effects. Grade 3/4 side effects were observed in neither group, and no patients withdrew due to side effects. Grade 2 dry skin and skin tenderness, and grade 1 liver disorder were seen in 1 patient each in the intervention group. Grade 2 liver disorder was seen in 1 patient in the control group.

## 4. Discussion

HU-induced leg ulcers, though rare, are recalcitrant to treatment and mandate cessation of HU therapy [21]. The present randomized trial showed that the Chinese medicinal herbal ZPO was effective in promoting the healing of HU-induced leg ulcers, providing an alternative treatment for the disease.

HU remains the therapeutic choice for myeloproliferative neoplasms such as polycythemia vera [19], essential thrombocythemia [22], and myelofibrosis [23]. Painful leg ulcers are a rare but troublesome complication that has been described in patients receiving high-dose, long-term HU treatment, often leading to discontinuation of HU. Although the exact mechanism for the development of this complication is still unclear, it has been hypothesized that inadvertent minor injury to the malleolar region that is inadequately repaired may jeopardize the regenerative potential of the epidermis. To date, only single case reports [14, 21, 24, 25] or single-center experiences [2, 26, 27] have been published regarding this phenomenon, with a reported incidence ranging from 5% to 10% of HU treated patients. A 5% incidence of skin ulcers during HU treatment also has been reported in a large randomized trial in patients with essential thrombocythemia [28]. Currently, the occurrence of skin ulcers during HU treatment is considered as HU intolerance [29].

Our data suggested that during the first three weeks, the two groups showed no statistically significant difference in the area healed (%), but by week 4, patients receiving ZPO exhibited a significant improvement compared to the control group. Moreover, all patients in the intervention group achieved complete healing at week 4. In addition, the intervention group achieved healing significantly more rapidly than the control group, with significantly fewer pathological scars, highlighting the efficacy of ZPO in healing HU-induced leg ulcers.

The underlying mechanism for the ulcer healing effects of ZPO still remains unclear. In chronic skin ulcer, intense and persistent inflammatory response, reduced angiogenesis and cell proliferation, decreased cell growth factors and collagen synthesis and degradation of excessive extracellular matrix all contribute to impairment of wound healing. Amongst these, the regeneration of capillary and vascular network formation plays an important role. As the most important regulatory factor of angiogenesis and neovascularization, vascular endothelial growth factor (VEGF) can stimulate the formation, proliferation, and migration of endothelial cells of the blood vessels [30]. The continuous secretion of VEGF plays a very important role in accelerating wound healing. Another study conducted by our team on the treatment of nonhealing wounds after breast cancer operation showed that the expression of VEGF in wound tissues was upregulated in patients treated with ZPO (data not shown).* Glycyrrhiza uralensis Fisch*, a component of ZPO, was reported to promote gastric ulcer healing by regulating inflammatory mediators [16]. We speculate that the ZPO may improve wound healing via multiple mechanisms including modulating the content of VEGF or inflammatory mediators. The levels of these molecules were not investigated in the current study and are worthy of further exploration.

Given the limited treatment options for HU-induced leg ulcers, our current randomized controlled trial demonstrated that the ZPO offers an effective therapeutic option for HU-induced leg ulcers. The study has certain limitations. The study population is relatively small, given the rarity of the condition. In addition, our hospital is a tertiary care center and the findings of the study may not be applicable to primary care settings. Furthermore, a mixture of medicinal herbs was used in the ZPO, rendering it difficult to attribute the actions of the ointment to any particular compound.

In conclusion, our randomized controlled trial demonstrates that the ZPO is effective in promoting the healing of HU-induced leg ulcers in patients with myeloproliferative neoplasms, providing a therapeutic option for a condition that is recalcitrant to conventional therapy.

## Figures and Tables

**Table 1:** The components of the Zhangpi Ointment and their pharmacological properties.

**Herbal components**	**Chinese names**	**Plant part used**	**Category by TCM theory**	**Phytochemical constituents**	**Pharmacological action in wound healing**
*Rheum officinale Baill*	Dahuang	Root	Purging drug	Rhein, emodin, chrysophanol, chrysaron, aloe-emodin, physcion	Hemostatic function, anticoagulation, inhibition of bacteria, anti-inflammation, antioxidant, reducing swelling and pain reduction
*Arnebia euchroma (Royle) Johnst.*	Zicao	Root	Heat-clearing drug	Shikonin, acetylshikonin, *β*-hydroxyisovalerylshikonin, teracrylshikonin	Anti-inflammation, inhibition of bacteria
*Angelica sinensis (Oliv.) Diels*	Danggui	Root	Blood tonifying drug	Ferulic acid, succinic, nicotinic acid, butylidenephthalide, folinic acid	Inhibition of platelet aggregation, anti-thrombosis, analgesic action
*Glycyrrhiza uralensis Fisch*	Gancao	Root	Heat-clearing drug	Glycyrrhizic acid, liquiritin	Detoxification, anti-inflammation
*Rehmannia glutinosa Libosch*	Shengdihuang	Root	Heat-clearing drug	*β*-sitosterol, mannitol, stigmasterol, campesterol, rehmannin	Detoxification, anti-inflammation
*Cortex Lycii*	Digupi	Root	Heat-clearing drug	Cinnamic acid, betaine, kukoamine A, lyciumin A, lyciumin B, *β*-sitosterol, linoleic acid, linolenic acid	Detoxification, anti-inflammation
Rubber powder	Xiangpifen	-	Wound healing	Myosin, actin, tropomyosin, hemoglobin, creatine, palmitic acid	Hemostatic function, promoting wound healing, detoxification, astringent effect
Calomel	Qingfen	-	Wound healing	Mercurous chloride (Hg_2_Cl_2_)	Astringent effect, hemostatic function, detoxification, inhibiting bacteria

**Abbreviations: **TCM, traditional Chinese medicine.

**Table 2:** Baseline characteristics of the patients.

**Characteristic**	**Total (n=49)**	**ZPO ( n=23)**	**Control ( n=26)**
Age (years), median and range	44 (24-60)	45 (24-60)	44 (29-60)
Gender			
Male	30 (61.2%)	12 (52.2%)	18 (69.2%)
Female	19 (38.8%)	11 (47.8%)	8 (30.8%)
Diagnosis			
Essential thrombocythemia	35 (71.4%)	16 (69.6%)	19 (73.1%)
Polycythemia vera	9 (18.4%)	5 (21.7%)	4 (15.4%)
Myelofibrosis	5 (10.2%)	2 (8.7%)	3 (11.5%)
Duration of ulcer at enrolment (months), median and range	10 (2-36)	9 (2-18)	11 (2-36)
BMI (kg/m^2^)			
<18.5	12 (24.5%)	4 (17.4%)	8 (30.8%)
18.5-24.9	29 (59.2%)	16 (69.6%)	13 (50.0%)
≥25	8 (16.3%)	3 (13.0%)	5 (19.2%)
Treatment after withdrawal of HU			
Recombined human interferon *α*	37 (75.5%)	16 (69.6%)	21 (80.8%)
Watchful waiting	12 (24.5%)	7 (30.4%)	5 (19.2%)
Routine blood test			
Leukocyte (×10^∧^9/L)	7.6±5.2	8.8±5.4	6.6±4.8
Hemoglobin (g/L)	115±36.3	116±36.9	114±37
Platelet (×10^∧^9/L)	303±179	298±167	308±183
Ulcer area (cm2)	3.5±1.0	3.3±1.0	3.6±1.0
Wagner's classification of the ulcers			
Grade 1	13 (26.5%)	5 (21.7%)	8 (30.8%)
Grade 2	21 (42.9%)	9 (39.1%)	12 (46.1%)
Grade 3	15 (30.6%)	9 (39.1%)	6 (23.1%)
Germiculture of the ulcers			
Positive	9 (18.4%)	6 (26.1%)	3 (11.5%)
Negative	40 (81.6%)	17 (73.9%)	23 (88.5%)
Pain evaluation (NRS classification)			
0-3	7 (14.3%)	3 (13.0%)	4 (15.4%)
4-6	28 (57.1%)	15 (65.3%)	13 (50.0%)
7-10	14 (28.6%)	5 (21.7%)	9 (34.6%)

**Abbreviations:** ZPO, Zhangpi Ointment; BMI, Body Mass Index; HU, hydroxyurea; NRS, Numerical Rating Scale.

**Table 3:** Adverse events in the study population.

	Intervention (%)				Control (%)			
	Grade 1	Grade 2	Grade 3	Grade 4	Grade 1	Grade 2	Grade 3	Grade 4
Cutaneous								
Allergy	0(0.00)	0(0.00)	0(0.00)	0(0.00)	0(0.00)	0(0.00)	0(0.00)	0(0.00)
Infections	0(0.00)	0(0.00)	0(0.00)	0(0.00)	1(3.85)	0(0.00)	0(0.00)	0(0.00)
Bleeding	1(4.35)	0(0.00)	0(0.00)	0(0.00)	1(3.85)	1(3.85)	0(0.00)	0(0.00)
Dry skin	0(0.00)	1(4.35)	0(0.00)	0(0.00)	1(3.85)	0(0.00)	0(0.00)	0(0.00)
Skin tenderness	1(4.35)	1(4.35)	0(0.00)	0(0.00)	2(7.69)	0(0.00)	0(0.00)	0(0.00)

Non-cutaneous								
Renal disorders	0(0.00)	0(0.00)	0(0.00)	0(0.00)	0(0.00)	0(0.00)	0(0.00)	0(0.00)
Liver disorders	1(4.35)	0(0.00)	0(0.00)	0(0.00)	0(0.00)	1(3.85)	0(0.00)	0(0.00)

## Data Availability

The data used to support the findings of this study are available from the corresponding author upon request.

## Abbreviations

MPNs: Myeloproliferative neoplasms
HU: Hydroxyurea
ZPO: Zhangpi Ointment
VEGF: Vascular endothelial growth factor.
